# Conversion of adverse data corpus to shrewd output using sampling metrics

**DOI:** 10.1186/s42492-020-00055-9

**Published:** 2020-08-11

**Authors:** Shahzad Ashraf, Sehrish Saleem, Tauqeer Ahmed, Zeeshan Aslam, Durr Muhammad

**Affiliations:** 1 College of Internet of Things Engineering, Hohai University, Changzhou, Jiangsu, 210032 China; 2 Muhammad Nawaz Sharif University of Engineering & Technology, Multan, 66000 Pakistan; 3 Petroweld Kurdistan Region, Erbil, 42002 Iraq; 4 Pakistan Steel Mills Karachi, Karachi, 75200 Pakistan

**Keywords:** Classification, Machine learning, Spread subsampling, Class imbalance

## Abstract

An imbalanced dataset is commonly found in at least one class, which are typically exceeded by the other ones. A machine learning algorithm (classifier) trained with an imbalanced dataset predicts the majority class (frequently occurring) more than the other minority classes (rarely occurring). Training with an imbalanced dataset poses challenges for classifiers; however, applying suitable techniques for reducing class imbalance issues can enhance classifiers’ performance. In this study, we consider an imbalanced dataset from an educational context. Initially, we examine all shortcomings regarding the classification of an imbalanced dataset. Then, we apply data-level algorithms for class balancing and compare the performance of classifiers. The performance of the classifiers is measured using the underlying information in their confusion matrices, such as accuracy, precision, recall, and F measure. The results show that classification with an imbalanced dataset may produce high accuracy but low precision and recall for the minority class. The analysis confirms that undersampling and oversampling are effective for balancing datasets, but the latter dominates.

## Introduction

The output of data from various fields has increased enormously. Dataset classification is a unique data mining (DM) technique, whose objective is to determine the target class belonging to a specific object in an unknown class. The result of a classification algorithm is generally related to data characteristics. One example of such an algorithm is a support vector machine (SVM) [1], which possesses numerous special advantages in solving classification problems, such as low sample numbers, nonlinearity, and high-dimensional pattern recognition. Moreover, the classification accuracy of the minority class is often more valuable. In the case of imbalanced data, the majority class examples will have a greater influence on the classifier, causing its classification weight to be in favor of the majority class and then seriously affecting the classification hyperplane distribution. Thus, classification approaches should be improved at the algorithm or data level to solve the imbalanced classification of data, which is currently a common problem in the field of DM research. Organizations are keen to process collected data and derive constructive information that can support their decision making [2]. DM [3] aims to collect, organize, and process huge amounts of data to identify useful unseen patterns. The Internet, being a vital tool of communication and information, is offering exclusive benefits to educators and students. Classification is one of the significant application fields in DM wherein the instances (records) in a dataset are grouped in more than one class. The classification can be a success or failure in the pedagogical environment or classifying flowers in different types [4]. The classifier gains knowledge from a prearranged training dataset; henceforth, to classify the instances from the unseen dataset, the class imbalance problem appears in datasets with an exceedingly unfair ratio between the classes [5]. This factor poses challenges for data mining and classification processes. Classifiers trained with an imbalanced dataset tend to predict the majority class (frequently occurring) more than the minority class (rarely occurring) [6]. This is due to the fact that standard classifiers are designed to concentrate on minimizing the overall classification error regardless of the class distribution. Thus, the classifier cannot easily learn from the class with a fewer number of instances.

Attention has been focused on the classification of imbalanced data. In recent years, many researchers have examined classification algorithms based on imbalanced data. Study approaches to the classification of imbalanced data by the SVM are currently primarily divided into two categories: improvements of approaches at the algorithm and data levels. The weighted SVM of the penalty coefficient *C* is used at the algorithm level to control the various costs of the misclassification errors of various classes. The minority class is generally charged a high cost of error classification, and the majority class is charged a low cost of misclassification. In addition, AdaBoost algorithm, the integrated multi-classifier algorithm and an enhancing kernel space-based algorithm, has been widely utilized. Two key approaches are present at the data level: oversampling of the minority specimens and undersampling of the majority specimens. The oversampling technique uses approaches to balance class distributions, such as the duplication of the minority example or artificial synthesizing of new minority class examples using certain algorithms. In addition to oversampling, undersampling is a common method of managing unbalanced datasets. In particular, undersampling balances the distribution of data classes with the elimination of majority class examples, such as the Tomek link algorithm [7].

The major contribution of this experimental research is to draw attention toward the misclassification issues, which result from training a classifier with a dataset where the instances in the class are not balanced, hereinafter collectively referred as the “imbalanced dataset”. This research clarifies that higher accuracy may not be enough to rank classifiers. This work proposes that classifiers’ performance can be enhanced with the implementation of sampling algorithms that eradicate the class imbalance problem. For the experiment, we consider a dataset from an educational institute where the majority of the attributes have real values [8].

In this study, we extracted underlying information from the confusion matrix and compared the classifier’s performance for the majority and minority classes. This analysis shows that accuracy may not appear as rigid evaluation criteria; rather, the focus should be on the classifier performance for minority and majority classes.

## Related work

Numerous solutions, either at the data or algorithm level, have been proposed to solve the class imbalance problem. At the data level, the proposed algorithms use various forms of re-sampling techniques, such as undersampling and oversampling. At the algorithm level, solutions include cost-sensitive learning, fine-tuning of the probabilistic estimation at the tree leaf (in decision tree implementation), adjusting the decision threshold, and preferring recognition-based learning rather than discrimination-based (in two-class) learning [9].

Educational DM [10] mines significant patterns in the data, collected from a pedagogical domain, to optimize the learner and learning environment. The classification models in a pedagogical environment forecast the learner’s expected academic outcome. Such a prediction model forecasts the final result (grade) of the student in a specific course. First, the model predicts the student with poor final grades. Then, the instructor intervenes to tutor the student and help him/her in achieving the improved final result. The limited number of students in a course leaves these datasets with a lower number of instances [11]. Moreover, a wide range of students’ attributes, such as attendance, marks in assessment tools, cumulative grade point average, credit hours, and marks in prerequisite courses, possess real values. The dataset in such environments suffers from class imbalance issues, wherein fewer learners have chances to perform unsatisfactorily. In this study, we consider a small imbalanced dataset, with attributes having nominal and real values, from a course in an institute.

In an empirical study, Pristyanto and Dahlan [12] demonstrated the use of oversampling and undersampling algorithms to improve the accuracy of instance selection methods on imbalanced databases. Their results yield that oversampling and undersampling methods improve accuracy. To improve the performance of classifiers based on emerging patterns, Sasikala et al. [13] used oversampling and undersampling methods. Similarly, Fatima and Mahgoub [14] implemented machine learning algorithms to classify students into binary classes (A and B). The dataset suffers from an imbalance ratio (IR), and the number of instances in class B is much bigger than that in class A. The results show that each of the applied algorithms has produced higher precision and recall for class B. Naïve Bayes classifier as the better-performing classifier yields a recall of 0.500 for class A and 0.851 for class B. All the implemented algorithms [naïve Bayes, multilayer perceptron (MLP), and decision tree] produced a higher recall, FP rate, and precision value for class B than class A. In addition, Kabakchieva made use of classification algorithms to classify students into five classes (excellent, very good, good, average, and bad) [15]. The dataset has over 4000 instances for the “very good” and ‘good’ classes and around 500 or less for the other three classes. The decision tree (J48) achieved recall values of less than 0.100 for ‘average’ and ‘excellent’ classes compared with other classes that achieved recall values of nearly or more than 0.70.

Some previous studies on class distribution and IR are presented in Table 1. Similarly, the difference in the performance evaluation of seven classes ranges from 0% to 83%, as shown in ref. [16]. Some results are evidence of the high diversity between the F measure of the majority and minority classes. The MLP has achieved the highest accuracy of 75%, but the difference between the F measure of the majority and minority classes is 0.244 (nearly one fourth). Similarly, it is nearly 50% in the case of SVM This finding draws attention toward the need for a proper class distribution before performing experiments to achieve reasonable results.

**Table 1:** Comparative analysis of previous work in relation to the class balancing ratio

Ref.	Class distribution/imbalance ratio					
Fatima and Mahgoub [14]	Class	A		B		
	Instances	62		195		
	Imbalance ratio	1		3.14		
Xie et al. [16] (Dataset-1)	Class	A	B	C	D	E
	Instances	2	22	38	8	2
	Imbalanced ratio	1	11	19	4	1
Xie et al. [16] (Dataset-2)	Class	A	B	C	D	E
	Instances	1	41	46	14	4
	Imbalanced ratio	1	41	46	14	4
Ashraf et al. [17]	Class	Excellent	Very good	Good	Average	Bad
	Instances	539	4336	4543	347	564
	Imbalanced ratio	1.55	12.5	13.10	1	1.60

Unbalanced classes are a common issue in the classification of machine learning, where the number of findings is disproportionated in class. Most algorithms for mastery learning work best if the sample numbers are approximately equal in each class [17]. Most algorithms have been developed to increase precision and decrease errors. Typically, the data imbalance represents an uneven class representation in a dataset. The fact that some classes have a slightly greater number of instances in the training set than certain classes is a typical issue in actual life implementations. Such a difference is called a class imbalance. Methods of addressing imbalances are well known for classical models of machine learning. Sampling methods are the most straightforward and common approach. These methods work on the data itself (instead of the model) to increase its balance. Of note, oversampling [18] is widely used and proven to be robust.

## Methods

**Fig. 1:**
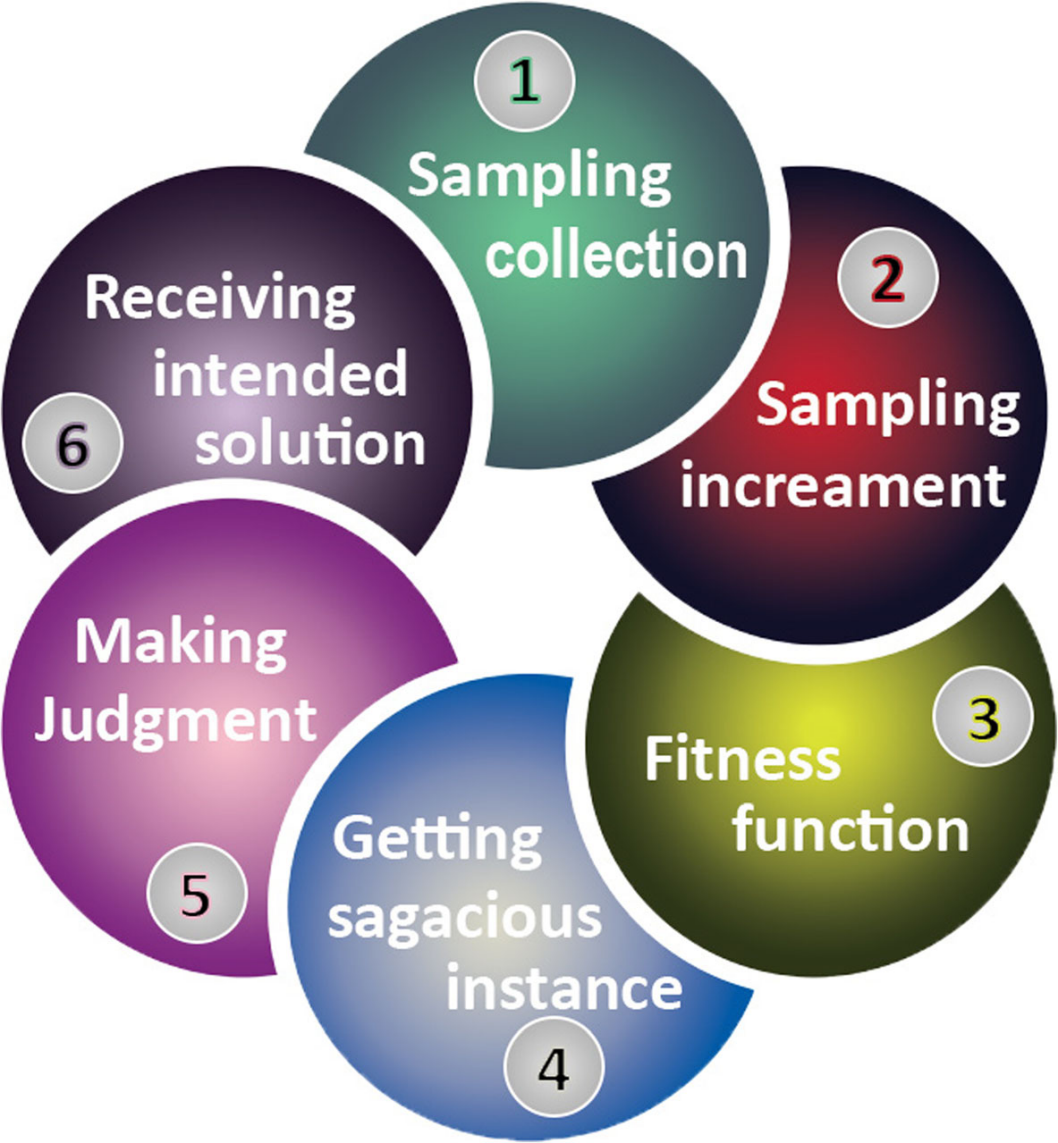
Information flow chart

The experiments have been performed in Waikato Environment for Knowledge Analysis (WEKA) [19]. WEKA, acknowledged as a landmark system in machine learning and DM, has become a widely used tool for DM research [20]. Classifier training is performed using a 10-fold cross-validation [21]. To select classifiers, we first categorized them and then selected one from each of the categories, probably the one found frequently in the literature. The findings are elaborated through a data flow diagram, shown in Fig. 1. Initially, from the data corpus, the samples were collected on the basis of the problem stated. All samples were applied in accordance to the mechanism described in the classification with the imbalanced dataset [22]. Each sample was incremented according to the required capacity. To balance the accuracy and manage the generated attributes that lead to new samples, the evaluation of the fitness function was performed. The fitness feature was also calculated based on the number of generations made to prevent an overfitted classification model [23]. When the criteria are accomplished, the final instances will be achieved; otherwise, the operators, such as selection, crossover, and mutation, will be utilized, and a balanced condition will be attempted to maintain by deriving substantial increments [24].

### Memory-based classifiers

In memory-based classifiers, the classification is directly based on the training examples. It stores the training set in the memory and then compares each instance with the instances in the training process. k-nearest neighbors (k-NN) [25] is an example of memory-based classifiers that plots each instance as a point in a multi-dimensional space and classifies it based on the class of their nearest neighbors.

### Artificial neural network

This computational model is inspired by the structural and functional characteristics of the biological nervous system. The MLP [26] is a class of artificial neural networks.

### Bayesian statistics

Bayesian inference is a method of statistical inference [27] based on using some evidence or observations in calculating the probability that a hypothesis may be true or update its previously calculated probability [28].

### SVMs

The SVM is a set of interrelated supervised learning methods that examine data and identify the patterns. Generally, naïve Bayes and SVM algorithms are considered better choices for text classification [29].

### Decision tree

Decision tree [30] is a recursive technique that builds a tree. It starts with a root node, probably the most important attribute, branching all the way through intermediate nodes and stopping at the end node.

### Performance metrics

The confusion matrix, precision, recall, and F measure were used to record the overall performance. Table 2 provides a standard visualization of a model with two class labels.

**Table 2:** Representation of the standard confusion matrix

	Positive	Negative
Positive	True positive	False negative
Negative	False positive	True negative

The ‘high’ class is considered a positive term and the ‘low’ class as a negative term. The rest of the terms are explained as follows: (1) True positive (TP): Predicted as ‘high’, and in actual fact, it is also ‘high’; (2) True negative (TN): Predicted as ‘low’, and in actual fact, it is also ‘low’; False positive: Predicted as ‘high’, but actually it is ‘low’; False negative: Predicted as ‘low’, but actually it is ‘high’.

- Recall

Recall is also called sensitivity or TP rate [31]. It is a measure of all positive instances and the number of instances that the model predicted correctly. It is the ratio of positive instances that are predicted correctly and the actual number of positive instances that can be calculated, as shown in Eq. 1.

1 $$ \mathrm{Recall}=\mathrm{TP}/\left(\mathrm{TP}+\mathrm{FN}\right) $$

- Precision

It shows all the positive instances that the model has predicted correctly [32] and the actual number of positive instances, as expressed by Eq. 2.

2 $$ \mathrm{Precision}=\mathrm{TP}/\left(\mathrm{TP}+\mathrm{FP}\right) $$

- F measure

The recall and precision values indicate the quality of the prediction model. However, making a decision based on the precision and recall values is sometimes not easy. The F measure takes precision and recall values into account and calculates their weighted average [33]. It is given in Eq. 3.

3 $$ \mathrm{F}\hbox{-} \mathrm{Measure}=2\times \left[\left(\mathrm{Precision}\times \mathrm{Recall}\ \right)/\left(\mathrm{Precision}+\mathrm{Recall}\right)\right] $$

- Accuracy

It is the ratio of the sum of the TP and TN and the total number of instances [34], as expressed in Eq. 4.

4 $$ \mathrm{Accuracy}=\frac{\left(\mathrm{TP}+\mathrm{TN}\right)}{\mathrm{n}} $$

where *n* is the total number of instance in the dataset.

### Dataset

The dataset contains 151 instances, which are the total number of students enrolled in the core course ‘CMP427’ during the three semesters taught in the IT Department at Hohai University, Changzhou, China. Final_Grade is the prediction feature with ‘low’ and ‘high’ classes. Usually, the students frequently obtaining grades below 65% are considered at risk of losing academic benefits.

#### Undersampling

This method is applied to the majority classes. Undersampling reduces the instances in the majority class to make them approximately equal to the instances in the minority class. Spread subsampling is one of the undersampling algorithms that we use in this research. Spread subsampling creates a random subsample of the imbalanced dataset. It adjusts the class distribution by randomly eliminating instances from the majority class [35]. To compute the distribution, spread subsampling takes a spread-distribution value (a parameter) from the user, which specifies the maximum ratio between the classes.

#### Oversampling

Synthetic minority oversampling technique (SMOTE) [36] oversamples the minority class with a random under sampling (RUS) of the majority class. This algorithm rebalances the original training set by conducting an oversampling approach. A SMOTE forms new instances for the minority class by interpolating among several minority class instances that recline together. The k-NN of the minority class instances are computed, and afterward, certain neighbors are selected. New synthetic data samples are generated from these neighbors [37]. SMOTE does not change the number of instances in the majority class; rather, it has a parameter (percentage) that specifies the desired increase in the minority class.

### Understanding oversampling and undersampling at the algorithm level

The SMOTE is an oversampling technique that synthetically produces instances by arbitrarily selecting minority class instances and using interpolation methods to produce instances between the selected point and its neighboring instances. Through this process, any instance of a minority class is considered, and new instances of a minority class are created along the line segment joining its nearest neighbors. The number of synthetic instances is generated based on the requisite percentage of oversampling. The algorithm steps are as follows: (1) Load data collection and classify the division of minority and majority classes; (2) Calculate the number of instances to be generated using the oversampling percentage; (3) Identify a minority class random case and locate its closest neighbors; (4) Choose one of the closest neighbors and determine the difference between the random instances and neighbors selected; (5) Multiply the difference by a number generated at random between 0 and 1; (6) Add that difference to the instance selected at random; and (7) Repeat the cycle from 3 to 6 until it produces the number of instances according to the percentage given.

Furthermore, RUS is a simple undersampling strategy that randomly excludes instances from the main class of the dataset before the classification methodology is applied. The main challenge of this strategy is that it can exclude relevant details in the dominant class that may not be appropriate in certain situations. The algorithm steps are as follows: (1) Launch the dataset and classify the minority and majority classes; (2) Calculate the number of instances to be removed on the basis of the percentage of undersampling; (3) Identify a random instance in the majority class and delete it from the majority class; and (4) Repeat step 3 until the number of instances eliminated is equal to the specified percentage.

## Results

**Fig. 2:**
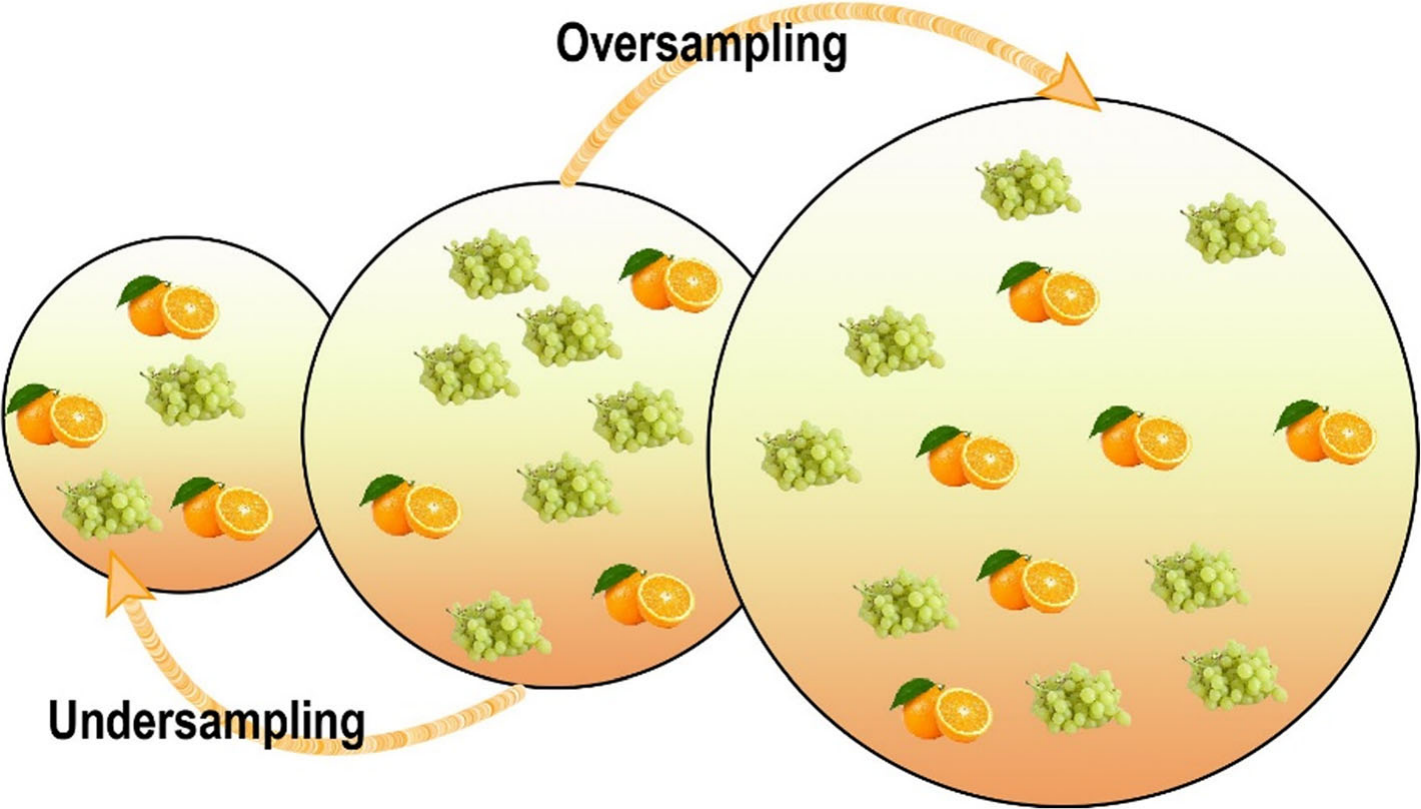
Depiction of oversampling and undersampling

Most of the classifiers tend to maximize accuracy despite a higher accuracy. A classifier may produce inadequate results, given that the training dataset is imbalanced. In an ideal dataset, the number of instances in the classes is more or less equal. The IR expresses how imbalanced a dataset is and is defined as the ratio of the sizes of the majority and minority classes. The dataset with IR = 1 is absolutely balanced, and thus the dataset with a higher IR is more imbalanced. Imbalanced classes bias the classifiers, which tend to classify all instances into the majority class. Data balancing refers to decreasing the value of IR and bringing it close to 1. The preceding literature shows that tuning class distribution can improve classifier performance. However, there is no unified rule for class balancing, but classification with sampling techniques yielded more optimal results than that without sampling techniques. Over time, a number of algorithms have been developed to deal with the class imbalance problem. The data-level algorithms make use of sampling techniques to adjust the IR. They are grouped as an oversampling or undersampling algorithm. Oversampling methods increase the number of instances in the minority class to balance the classes; by contrast, undersampling remove instances from the majority class to adjust the class distribution. Figure 2 depicts the idea of undersampling and oversampling algorithms. The center dataset is imbalanced with gapes in the majority class; the left side illustrates the dataset after undersampling where instances are removed from the grapes class, whereas the orange instances are added to the right side when oversampling is performed.

### Classification with imbalanced datasets

**Fig. 3:**
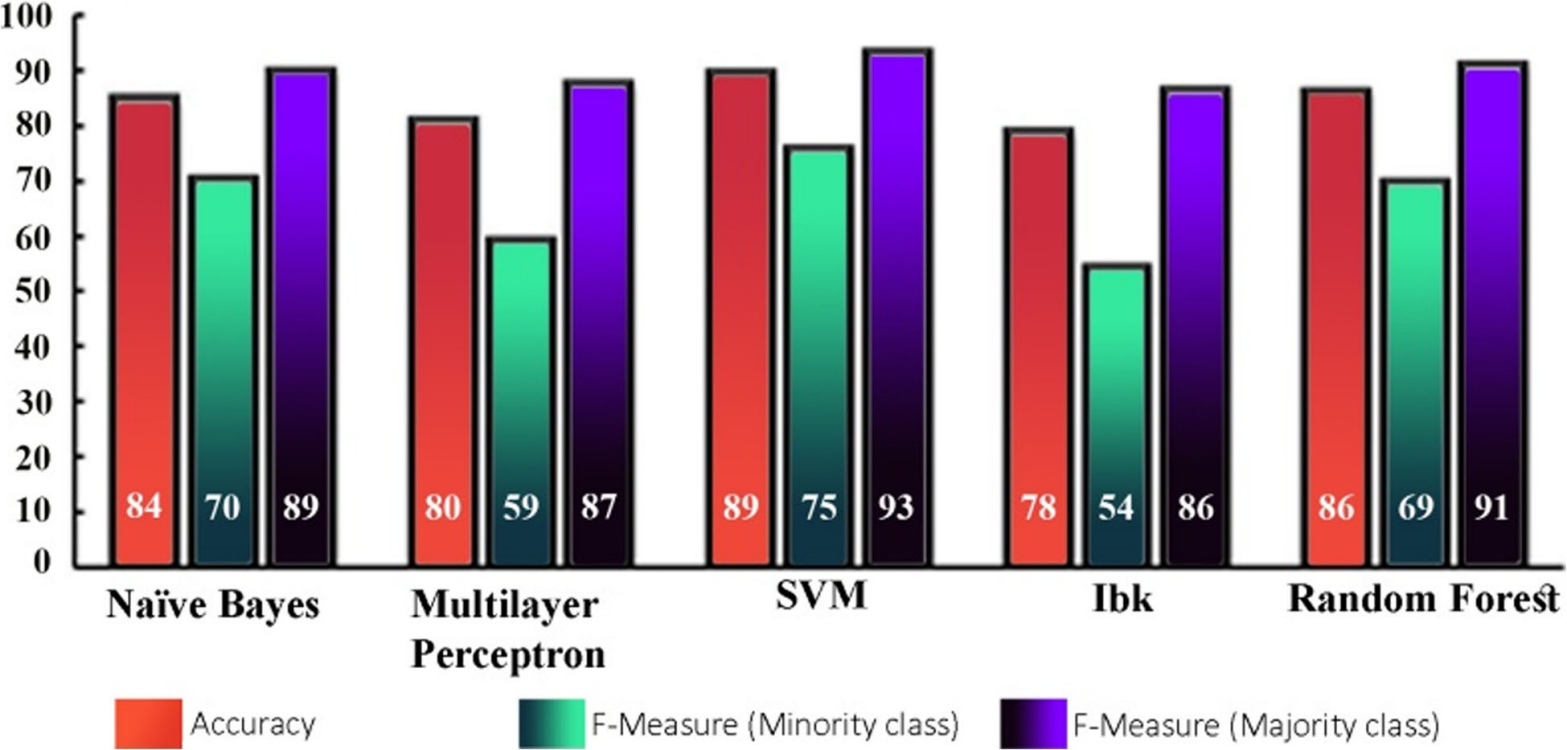
Accuracy comparison and the F measures of classifiers for the minority and majority classes over the imbalanced dataset

We acquired an imbalanced dataset from an educational environment with an IR = 1:3.19. Around two-thirds of the instances were from the majority (high) class compared to the low number of instances in the minority (low) class. We performed classification with an imbalanced dataset to compare the accuracy of the classifiers with other performance evaluation measures. Table 3 shows the results obtained from the classifiers. It outlines the accuracy of each classifier and provides the precision, recall, and F measure for minority (low) and majority (high) classes and also their average. The last column provides the confusion matrix for each classifier. The results show that most of the classifiers have produced more than 80% accuracy. The confusion matrix identifies the number of instances in each class that are misclassified by each classifier. Figure 3 shows a chart that compares the accuracy (data labels at the top of the bar) of each classifier and the F measure (in percent) for the minority (data labels at the center of the bar) and majority (data labels at the bottom of the bar) classes. Despite achieving higher accuracies and F measures for the majority class, the classifiers have achieved relatively lower F measure values for the minority class. For instance, SVM has an exceptionally low F measure for the minority (75.8%) class but high accuracy (89.4%). Moreover, the difference between the F measure of the majority and minority classes is high for all classifiers. It concludes the bias behavior of classifiers over an imbalanced dataset. The classifiers achieved reasonably high accuracy but failed to correctly classify the minority class instances.

**Table 3:** Results of classification with the imbalanced dataset

Classifier	Accuracy	Classes	Precision	Recall	F-Measure	Confusion matrix
Naïve bayes	84.77%	Low	0.659	0.750	0.701	a b < −- classified as 27 9 | a = Low 14,101 | b = High
		High	0.918	0.878	0.898	
		Average	0.856	0.848	0.851	
Multilayer perceptron	80.79%	Low	0.600	0.583	0.592	a b < −- classified as 21 15 | a = Low 14,101 | b = High
		High	0.871	0.878	0.874	
		Average	0.806	0.808	0.807	
SVM	89.40%	Low	0.833	0.694	0.758	a b < −- classified as 25 11 | a = Low 5110 | b = High
		High	0.909	0.957	0.932	
		Average	0.891	0.894	0.891	
IBk	78.81%	Low	0.559	0.528	0.543	a b < −- classified as 19 17 | a = Low 15,100 | b = High
		High	0.855	0.870	0.862	
		Average	0.784	0.788	0.786	
Random forest	86.09%	Low	0.727	0.667	0.696	a b < −- classified as 24 12 | a = Low 9106 | b = High
		High	0.898	0.922	0.910	
		Average	0.858	0.861	0.859

### Undersampling dataset classification

**Fig. 4:**
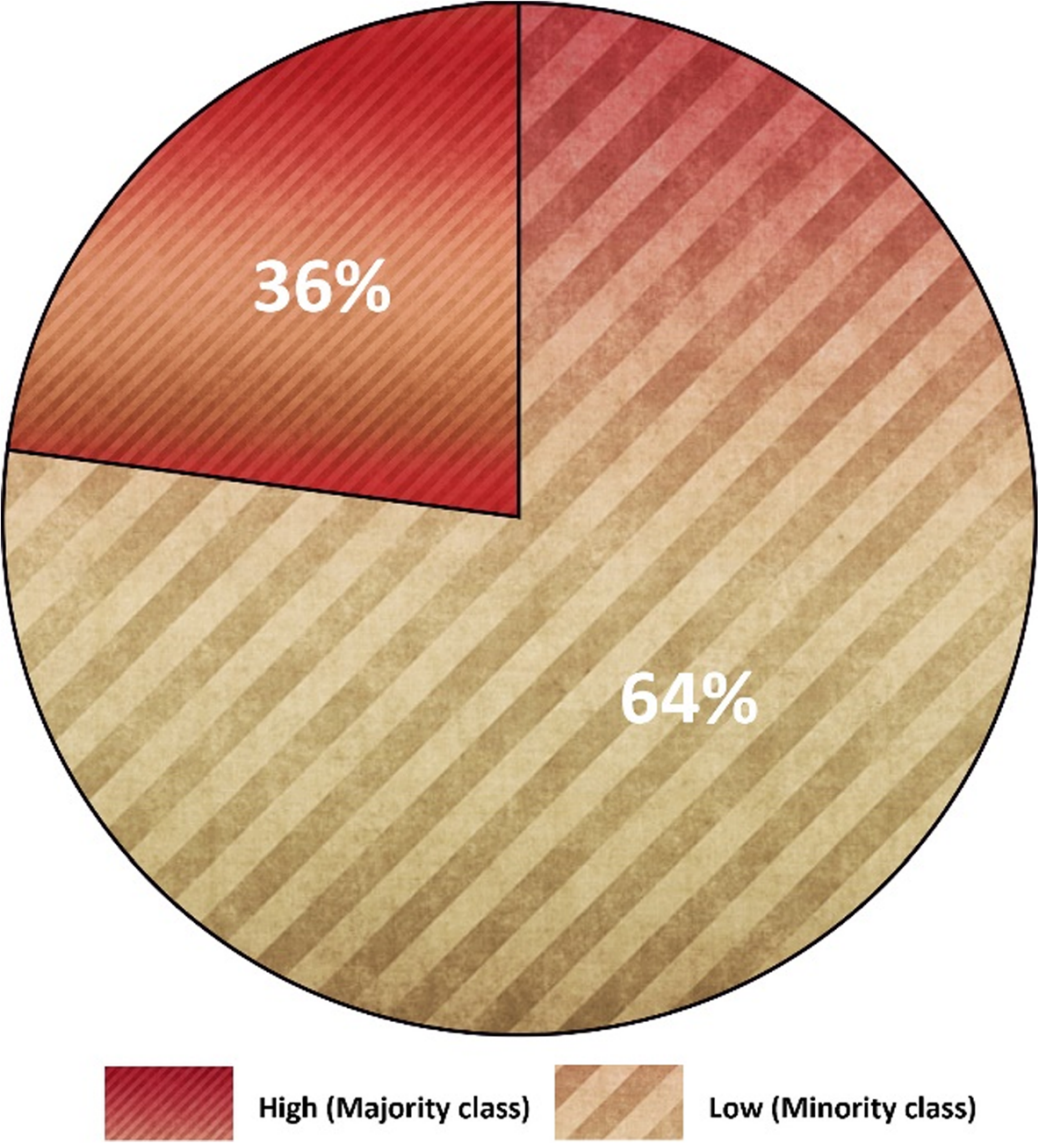
Imbalanced dataset

**Fig. 5:**
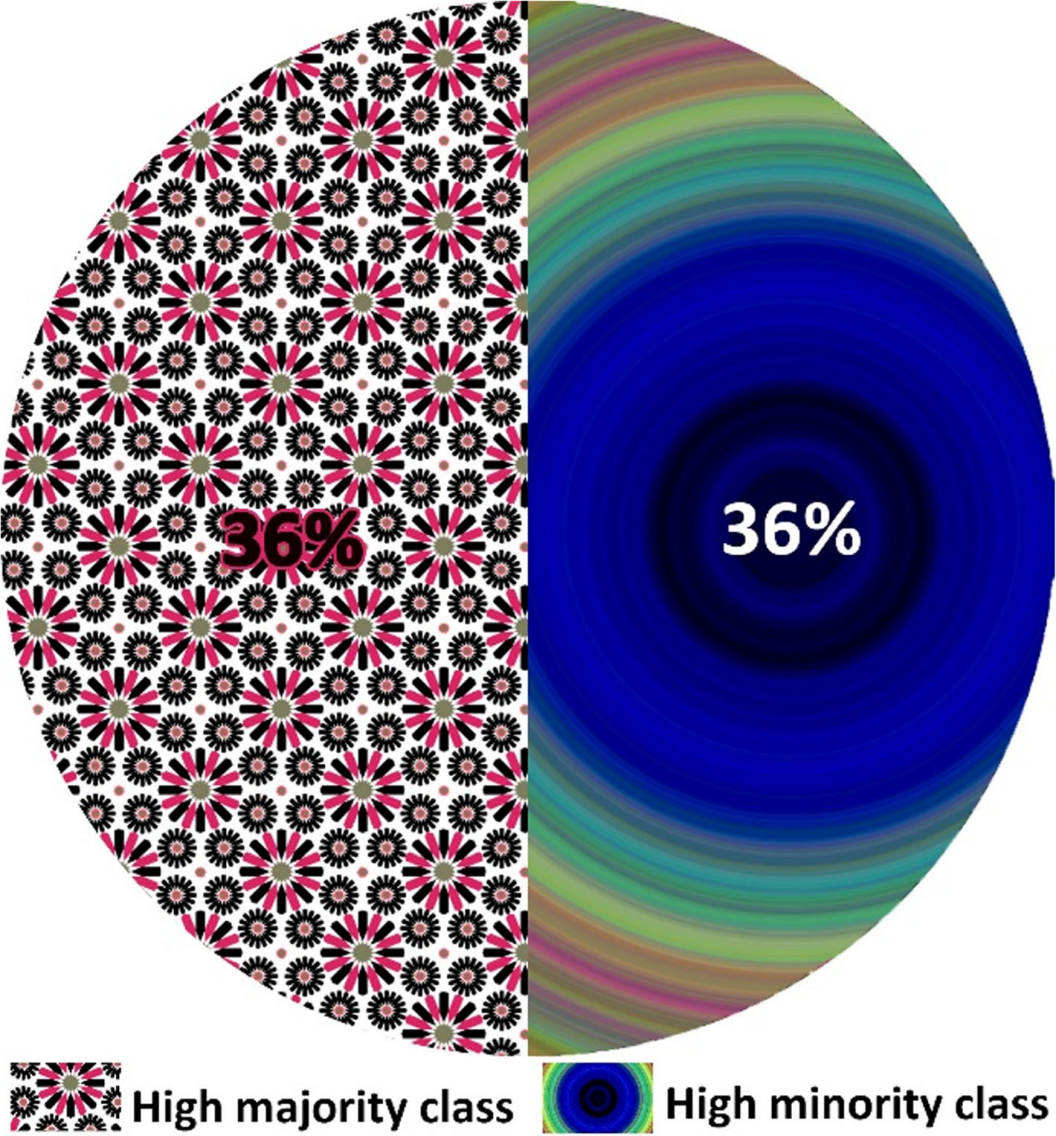
Balanced dataset

We applied spread subsampling algorithm, an undersampling algorithm for balancing the imbalanced dataset. Figures 4 and 5 illustrate the impact of spread subsampling-produced datasets. Similarly, Table 4 shows the performance measures of classifiers when spread subsampling was implemented. SVM and MLP achieved the highest accuracy. MLP achieved slightly higher F measure and recall values for the minority class. The confusion matrix shows that MLP misclassified only four instances of the minority class compared with the SVM that misclassified five.

**Table 4:** Classification results after oversampling

Classifier	Accuracy	Classes	Precision	Recall	F-Measure	Confusion matrix
Naïve bayes	87.89%	Low	0.852	0.907	0.879	a b < −- classified as 98 10 | a = Low 17 98 | b = High
		High	0.907	0.852	0.879	
		Average	0.881	0.879	0.879	
Multilayer perceptron	91.03%	Low	0.873	0.954	0.912	a b < −- classified as 103 5 | a = Low 15,100 | b = High
		High	0.952	0.870	0.909	
		Average	0.914	0.910	0.910	
SVM	88.79%	Low	0.849	0.935	0.890	a b < −- classified as 101 7 | a = Low 18 97 | b = High
		High	0.933	0.843	0.886	
		Average	0.892	0.888	0.888	
IBk	83.86%	Low	0.805	0.880	0.841	a b < −- classified as 95 13 | a = Low 23 92 | b = High
		High	0.876	0.800	0.836	
		Average	0.842	0.839	0.838	
Random forest	90.13%	Low	0.898	0.898	0.898	a b < −- classified as 97 11 | a = Low 11,104 | b = High
		High	0.904	0.904	0.904	
		Average	0.901	0.901	0.901	

**Fig. 6:**
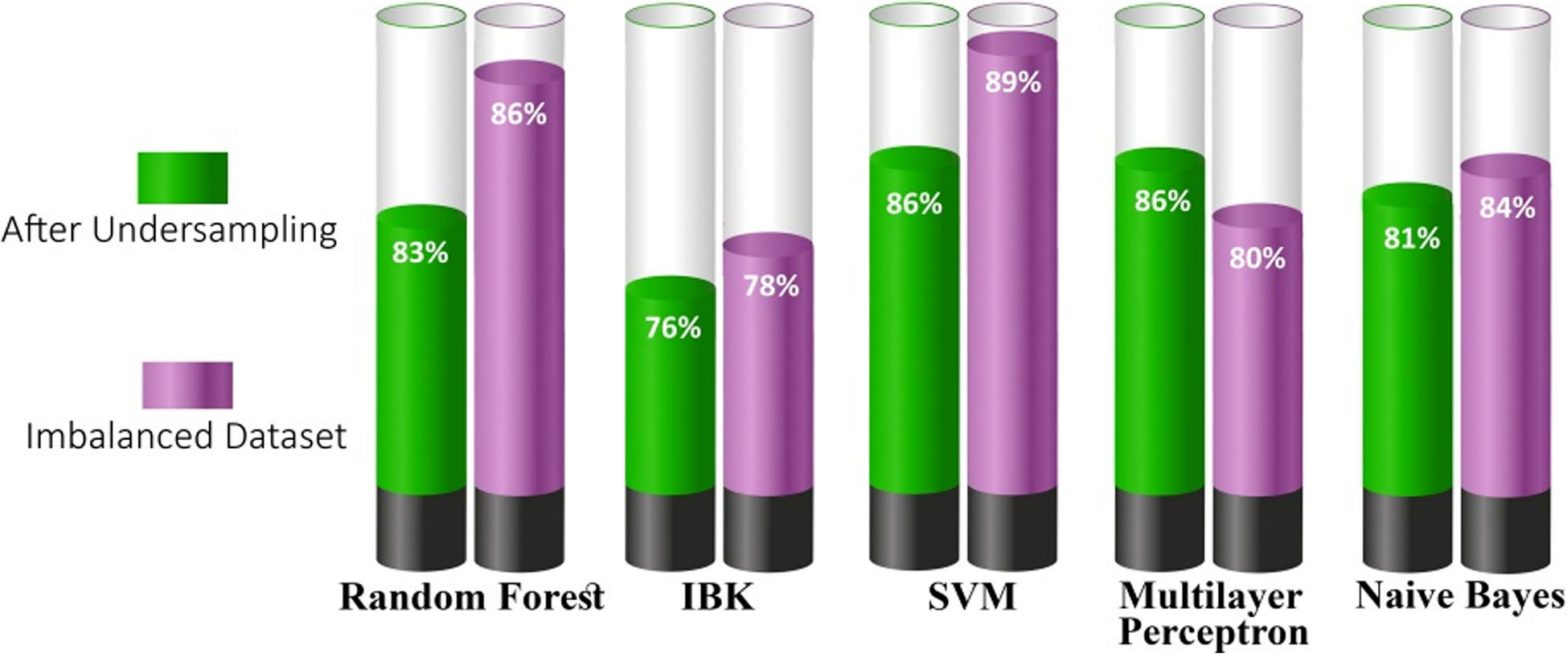
Performance comparison before and after undersampling

To compare the classification with the imbalanced and undersampled datasets, a specified chart is illustrated in Fig. 6, which presents the decrease in the accuracy of classifiers (except MLP) after undersampling. This finding may indicate that the classifiers have reduced partiality and have properly classified instances.

### Oversampling dataset classification

**Fig. 7:**
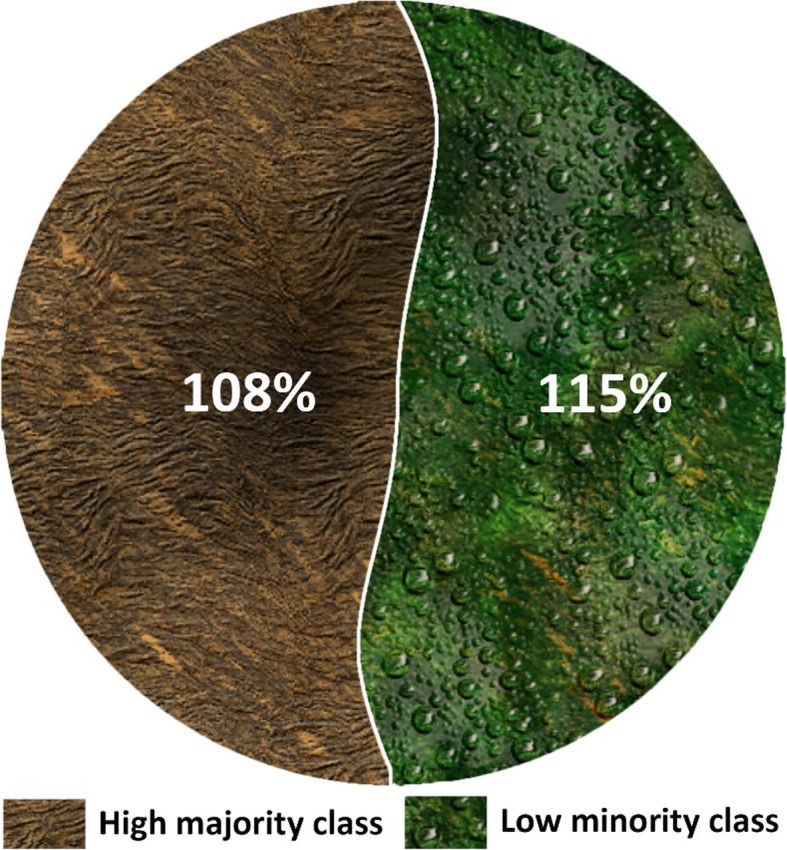
Class distribution after oversampling

**Fig. 8:**
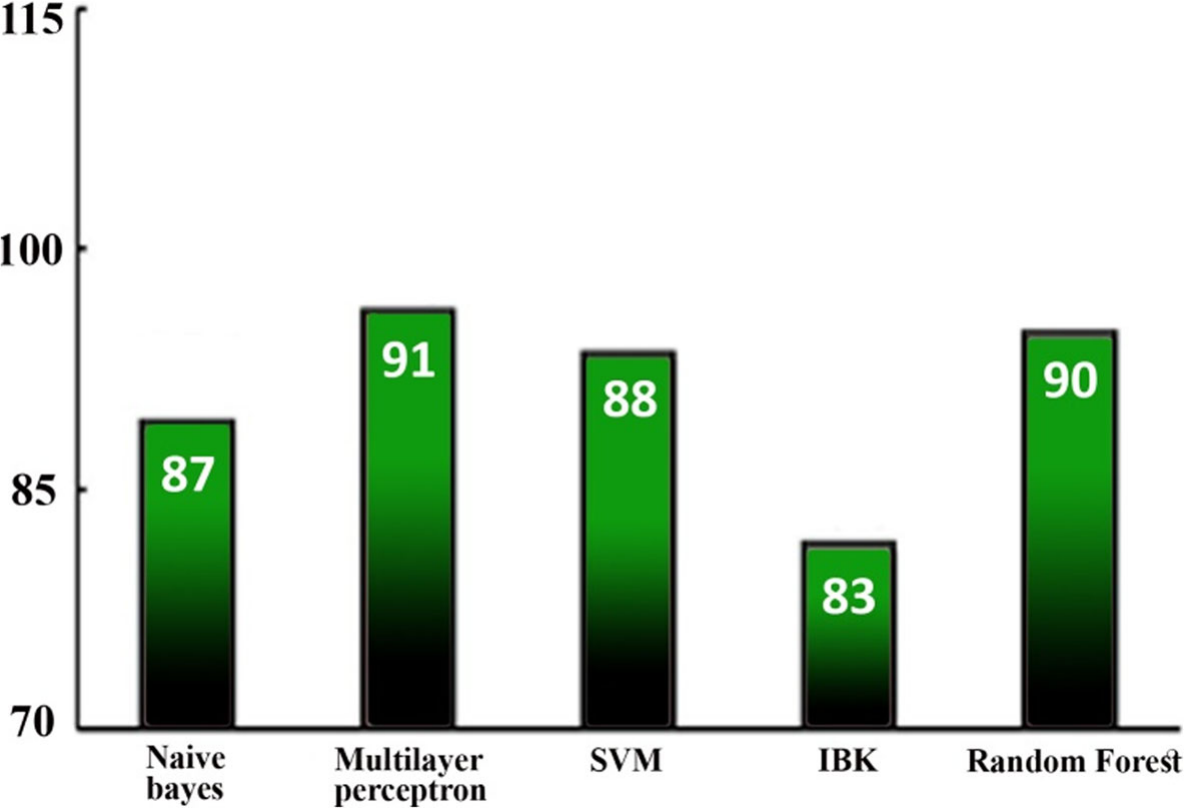
Performance comparison using the average F measure

**Fig. 9:**
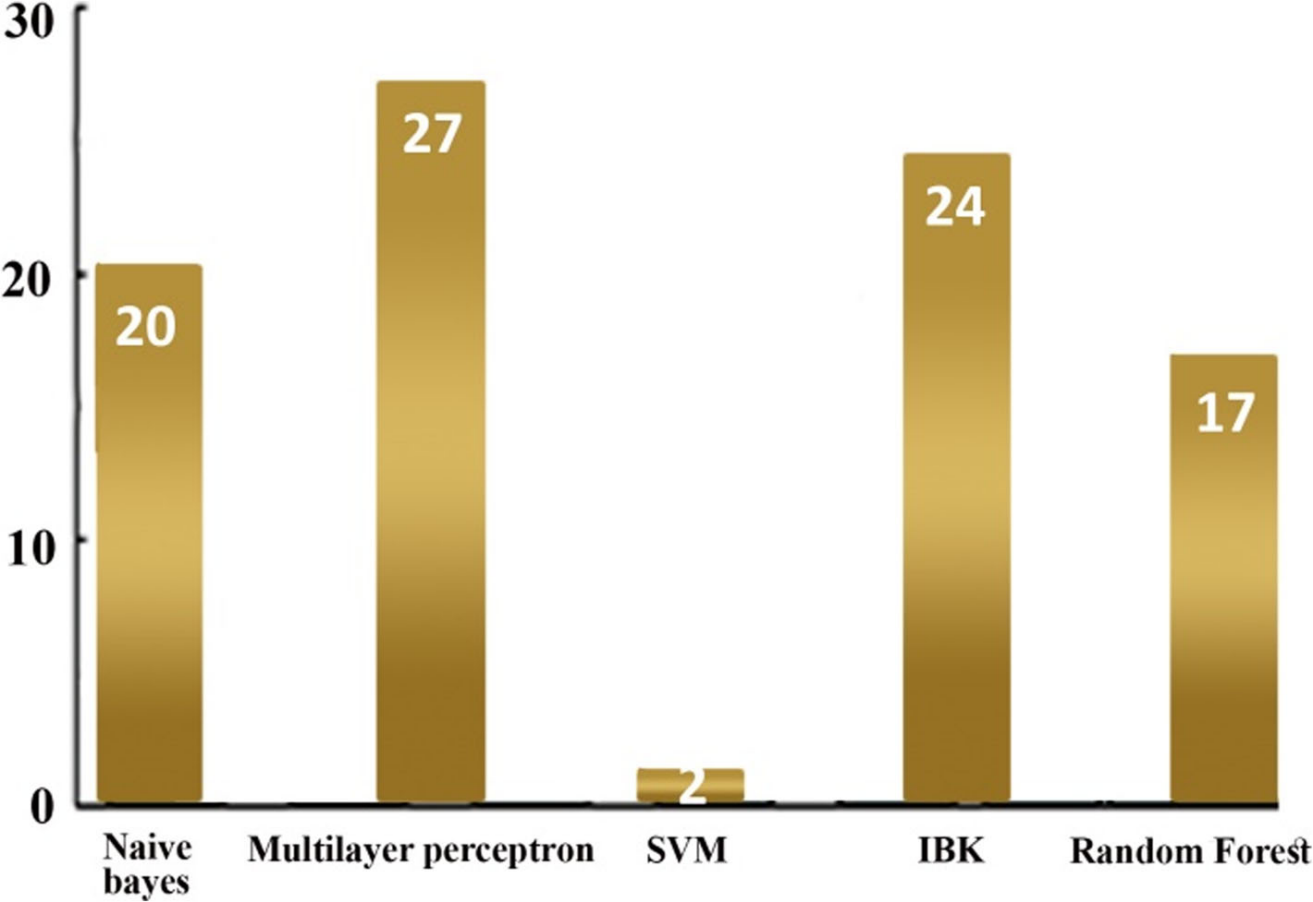
Precision increase with oversampling

SMOTE has been utilized to balance datasets through oversampling. With 200 as the percentage value, SMOTE approached 108 instances of the minority class. Figure 7 shows the class distribution after oversampling. SMOTE appends the newly created instances at the end of the dataset file. The use of k-fold cross-validation will possibly give rise to data overfitting. To avoid overfitting, we randomized the instances in our dataset. Table 4 provides the results for the classification after oversampling. The application of SMOTE has further enhanced the performance of the classifier. MLP has achieved the highest accuracy. The chart in Fig. 8 compares the classifiers’ performance using the average F measure after oversampling. This chart confirms that the average F measure for classifiers has increased with oversampling for both datasets. The chart in Fig. 9 highlights an increase in the precision (in percent) of the minority class with oversampling. This chart illustrates that oversampling has increased the precision of the minority class. The highest increase was achieved by MLP, and the lowest was achieved by SVM.

## Conclusions

The outcome shows that not only the accuracy of a classifier decides whether it is predicting well. In fact, other performance measures, such as F measure, precision, and recall values for the minority class, should be observed as well. This supports the argument that classifiers with an imbalanced dataset tend to misclassify most of the instances as the majority class. We observed that undersampling and oversampling algorithms are effective in decreasing the difference between the F measures of the majority and minority classes. In both cases, the classifiers achieved reasonable accuracies and F measure values. However, between the two sampling algorithms, oversampling (SMOTE) performed better than undersampling. The oversampling approach shows superiority over undersampling SMOTE.

The comparative analysis with oversampling and undersampling algorithms was conducted for classifiers with imbalanced datasets for the data collection. The sample was drawn from a perspective in training. The classifier was split into various groups, and from each group, one classifier was chosen. We believe that classification with an imbalanced dataset will yield higher accuracy for the minority class but low F measure values. Hence, the classifiers misclassify cases of the minority class. In our dataset, we implemented undersampling (spread subsampling) and oversampling (SMOTE). The findings indicate that the F measure levels for the minority class are improved by spread subsampling and SMOTE. Nonetheless, SMOTE performs well in achieving a higher F measure value and accuracy than spread subsampling.

## Data Availability

For any relevant content, a request may be sent to the corresponding author.

## Abbreviations

DM: Data mining
EDM: Educational data mining
SVM: Support vector machine
MLP: Multilayer perceptron
IR: Imbalance ratio
WEKA: Waikato Environment for Knowledge Analysis
TP: True positive
FP: False positive
FN: False negative
TN: True negative
SMOTE: Synthetic minority oversampling technique
RUS: Random under sampling
